# Commenting on “Prognostic and diagnostic significance of copeptin in acute exacerbation of chronic obstructive pulmonary disease and acute heart failure: data from ACE 2 study” by Jacob A. Winther and colleagues

**DOI:** 10.1186/s12931-018-0738-7

**Published:** 2018-03-01

**Authors:** Luigi Mario Castello, Mattia Bellan

**Affiliations:** Translational Medicine Department, Università del Piemonte Orientale, Via Solaroli, 17, 28100 Novara, Italy

**Keywords:** Copeptin, Biomarkers, Prognostic factor, Dyspnea, Heart failure, Acute exacerbation of chronic obstructive pulmonary disease

## Abstract

We would like to comment on the article entitled “Prognostic and diagnostic significance of copeptin in acute exacerbation of chronic obstructive pulmonary disease and acute heart failure: data from ACE 2 study” by Jacob A. Winther and colleagues, in the light of the results of a multicentric study published in 2014 by Vetrone F. et al., in which 336 patients with dyspnea were enrolled in the Emergency Departments of three University Hospitals in Italy.

These two studies confirm the prognostic role of copeptin in patients with dyspnea due to heart failure but, while Winther et al. performed the copeptin measurements only at admission, Vetrone et al. evaluated the time-course of copeptin plasma concentration from the admission to the hospital discharge. The results showed a better performance of copeptin measured at discharge as prognostic biomarker compared to copeptin at hospital admission; similarly, a lower reduction or an increase in copeptin concentration from admission to discharge was a strong prognostic predictor of unfavorable outcome. In our opinion this is a very important result, opening new perspectives for the use of copeptin as prognostic marker in HF patients.

Dear Editor,

We read with interest the article entitled: “Prognostic and diagnostic significance of copeptin in acute exacerbation of chronic obstructive pulmonary disease and acute heart failure: data from ACE 2 study” by Jacob A. Winther and colleagues. The Authors evaluated the clinical usefulness of copeptin as a diagnostic tool and prognostic marker, in comparison to NT-proBNP, in a population of 314 patients admitted because of acute dyspnea at the Akersus University Hospital, in Norway [1].

Beside confirming that NT-proBNP better performs than copeptin in differentiating patients with heart failure (HF) related dyspnea from those complaining non-HF related dyspnea, the Authors outlined how promising copeptin is as prognostic biomarker. In fact, the major finding was that the two biomarkers show similar accuracy in predicting 2-years mortality in the group of HF patients. On the contrary, copeptin but not NT-proBNP, predicted mortality in acute exacerbation of chronic obstructive pulmonary disease (AECOPD). These results raised several issues which need to be further discussed at the light of the current literature dealing with this topic. Copeptin had already been evaluated as short and long-term prognostic marker in AECOPD, with conflicting results. Stolz et al. have reported that copeptin levels at admission are correlated with the length of the hospital stay; moreover copeptin was predictive for long-term clinical failure [2]. This finding is also supported by the results of Boeck et al., who proposed a simple score, inclusive of copeptin, which was predictive of 2-years mortality [3]. On the contrary, Dres et al. failed to disclose an association between copeptin plasma concentration and a short-term composite outcome (ER visit, death or transfer to the Intensive Care Unit) [4].

The prognostic value of copeptin in HF patients has been widely investigated and many studies suggest that elevated copeptin plasma concentration is a predictor of mortality in HF [5]. What is less clear and more debatable is the best moment for the performance of the dosage.

In a multicentric study published in 2014 by Vetrone F. et al., 336 patients with dyspnea were enrolled in the Emergency Departments (ED) of three University Hospitals in Italy; the major aim of the study was to evaluate the short term prognostic role of copeptin in patients divided in HF and non-HF groups. Copeptin was measured at the admission and at the discharge from the hospital and the follow-up time was 3 months. Copeptin at admission was validated as a short term prognostic factor; however, the original finding of this study, was the prognostic role of copeptin measured at discharge; similarly, a lower reduction or an increase in copeptin concentration from admission to discharge was a strong prognostic predictor of unfavorable outcome [6].

Although several differences do exist between these two studies, we think that some comparisons can be performed: first of all the median copeptin concentrations at enrollment (T0) measured by Vetrone et al. were higher with respect to those measured by Winther et al. (42 pmol/L vs 22.2 pmol/L respectively in HF patients and 20 pmol/L vs less than 9 pmol/L in patients with non HF-related dyspnea). These differences can be explained by the different setting of the enrollment, since Winther et al. set T0 within the first 24 h after the hospital admission while Vetrone et al. set T0 in an earlier phase, in the ED, before starting any treatment. Although copeptin is more stable with respect to arginine-vasopressin (AVP), its plasma concentration may vary after the first hours of treatment according to hemodynamic stabilization and drugs administration.

The second point of discussion is the importance of copeptin concentration at discharge, a relatively stable clinical phase during which a balance is obtained in most patients; in our opinion discharge copeptin concentration can be assumed as the “standard” of the patient and this value should be considered for the prognostic stratification. Looking at the receiver operating characteristic (ROC) curves presented by Vetrone et al., obtained pooling all the patients studied and considering as categorical variable the presence or absence of events (death or re-hospitalization) during the 3 months follow-up, the area under the curve (AUC) was 0.63 considering T0 copeptin concentration and 0.83 considering copeptin concentration at discharge from the hospital. In our opinion this is a very important result, opening new perspectives for the use of copeptin as prognostic marker in HF patients. It should be, however, stressed that in the Italian cohort, the follow-up was 3 months, significantly shorter than in Winther’s study. Therefore, the prognostic value of copeptin at discharge deserves a more specific evaluation for longer follow-up periods.

As emergency physicians we are used to divide the clinical path of patients affected by acute conditions in three stages: in the first one, the ED approach, the priorities are to rule out life threatening conditions, to perform a differential diagnosis and to stabilize the patient. This latter can be obtained in the ED, with a short stay in the observation unit or may require hospitalization. The next phases are the discharge from the hospital and the follow-up. We think that more data about the time course of copeptin plasma concentration in these clinical phases of different diseases (first of all HF) could be useful in order to contribute to identify high-risk patients requiring hospitalization or a closer follow-up.

## Abbreviations

AECOPD: Acute exacerbation of chronic obstructive pulmonary disease
AUC: Area under the curve
AVP: Arginine-vasopressin
HF: Heart failure
ROC: Receiver operating characteristic
